# New Treatment of Burns

**Published:** 1874-09

**Authors:** M. Olmstead Baldwin


A NEW TREATMENT FOR BURNS.—*By M. Olmstead Baldwin, M.D.* * * * For a considerable time it has been our custom to use eggs, the white and yolk together, well beaten up, as a local application, and the remedy has given a great degree of satisfaction. Our manner of applying is, after the eggs are well beaten, to saturate old and well-worn pieces of muslin therein, and spread over the injured surface, two or three layers thereof being superimposed; the relief is immediate and complete. The dressing should be renewed each twelve hours; meantime, should it become dry, it may be moistened by dripping water over it. After the first two or three dressings we add a little carbolic acid and glycerine, to correct any disagreeable odor, and also stimulate the healing process. The dressing is easily removed, and leaves a clean, fresh-looking surface, not attainable under the old processes.—*Chicago Medical Journal—August.*

From a letter dated London, June 10, 1874, received by a medical gentleman of this city, we learn that Mr. Erichsen is about to visit this country on a tour of pleasure and of observation. He will leave England on the 30th of July, and, after spending a few weeks in Canada, where he has friends and connections, he will pass into the United States, where, we are sure, he will everywhere meet with a cordial welcome from his professional brethren. As a surgical author, Mr. Erichsen has won for himself a world-wide reputation.—*Philadelphia Medical Times.*

